# Thermodynamic Study of the Corrosion of Refractories by Sodium Carbonate

**DOI:** 10.3390/ma11112197

**Published:** 2018-11-06

**Authors:** Ying Zhao, Guishi Cheng, Yu Xiang, Fei Long, Changqing Dong

**Affiliations:** 1 National Engineering Laboratory for Biomass Power Generation Equipment, School of the Renewable Energy, North China Electric Power University, Beijing 102206, China; 51101890@ncepu.edu.cn (G.C.); xiang_yu1991@163.com (Y.X.); cqdong@ncepu.edu.cn (C.D.); 2 Department of Mechanical and Material Engineering, Queen’s University, Kingston, ON K7L 3N6, Canada; long.fei@queensu.ca

**Keywords:** refractory, corrosion, thermodynamic, sodium carbonate

## Abstract

The corrosion of refractories by sodium salts in waste liquid at high temperature has become a serious problem. This paper focuses on the thermodynamic characterization of sodium carbonate (Na_2_CO_3_) corrosion of six refractories by FactSage modelling in combination with X-ray diffraction (XRD). Three of the refractories are oxides (Fe_2_O_3_, Al_2_O_3_, and Cr_2_O_3_), and the other three are synthetics spinels (magnesium chromium, MgO·Cr_2_O_3_; magnesioferrite, MgO·Fe_2_O_3_; and, magnesium aluminium, MgO·Al_2_O_3_). First, thermodynamic simulations were carried out with the FactSage thermodynamics model using the reaction package to predict the direction of the Na_2_CO_3_ corrosion reaction in terms of the Gibbs free energy. Then, the reactions between the six refractories and Na_2_CO_3_ were conducted through a series of refractories/Na_2_CO_3_ reaction tests. The XRD analytical method was used to describe and understand the chemistry and interpret mineral matter transformation. The products of the tests were also determined by X-ray diffraction and the experimental observations were compared with the results of the thermodynamic simulations. Furthermore, the strength of sodium corrosion of the refractory materials was comprehensively discussed. The results show that MgO·Al_2_O_3_ has the best thermal stability and it is hard to corrode by Na_2_CO_3_, while the chrome-containing refractory reacts easily with Na_2_CO_3_ with a considerably high amount of corrosion product at a temperature of 600 °C. These experimental results are in agreement with the thermodynamic calculations.

## 1. Introduction

With rapid industrialization, large amounts of waste are generated in production processes in China. The emission of organic waste liquids has risen sharply, which causes serious environmental pollution and harms human health. The incineration technology is an effective and feasible treatment for organic compounds, because chemical waste liquids have a calorific value. This approach can recover and utilize the heat that is contained in high-concentration waste liquid, reduce the treatment cost, as well as burn off harmful substances towards environmental protection.

Refractories are essential for high-temperature resistance equipment and are widely used in the electric power, iron and steel, cement, nonferrous metal smelting, ceramics, chemical, and other industrial fields. There are severe corrosion problems that are associated with refractories in steel refining furnaces [1], coal gasifiers [2,3], rotary kilns for the high-temperature calcination of cement [4], solid waste incinerators [5,6], liquid waste incineration boilers, and other industrial equipment.

**Figure 1:**
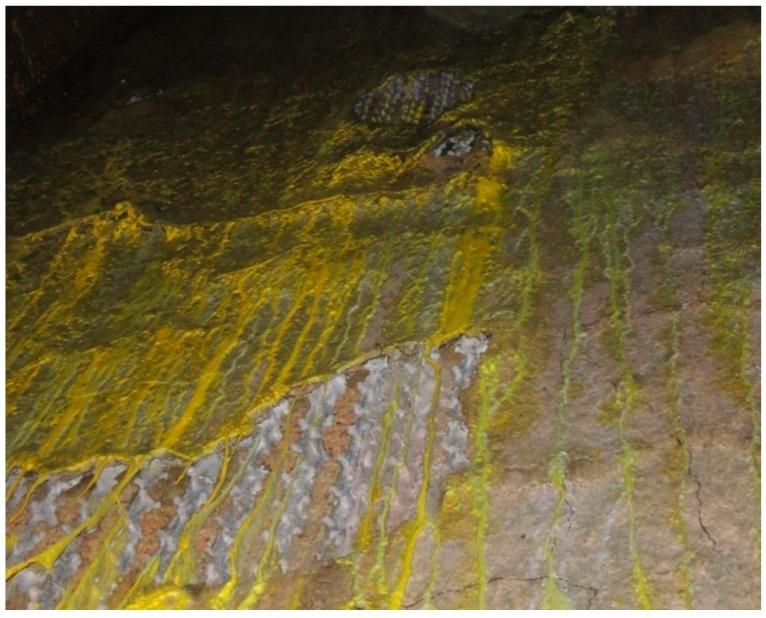
Corrosion of the refractory material by sodium salts in a waste liquid boiler.

Many studies found that refractories containing Cr_2_O_3_ have excellent resistance to slag corrosion [5,7,8,9,10]. Cr_2_O_3_ has low solubility in the slag and it can increase the viscosity of the molten slag, thus reducing the chemical erosion of the refractory. Moreover, the increase in viscosity reduces the permeability and wettability of the refractory material and the formation of the metamorphic layer, thereby reducing the flake refractory material structure. However, in waste liquid boilers, alkali salts can cause severe corrosion in refractories [11]. In addition, refractories are more prone to mechanical damage at high temperature; consequently, the service life of refractory materials might be shortened and great hidden danger and economic losses might occur. Figure 1 shows the corrosion of the refractory material of a sodium salt-containing waste liquid boiler. Hence, it is significant to study the corrosion of refractory materials by sodium salts.

A large number of scientists studied the influence of additives on the corrosion resistance of refractories. Some of the researchers found that additive could significantly improve the slag corrosion resistance [12,13,14,15,16]. Presently, new anti-sodium corrosion refractories have been studied by changing the ratio of refractory materials [17,18,19,20]. Weinberg et al. [6] investigated the corrosion of the Al_2_O_3_-SiO_2_ refractories of hazardous waste incinerators by sodium and sulfur vapours and found that the refractories should contain minimal amounts of silica to prevent these corrosion processes.

Many researchers used scanning electron microscopy (SEM), energy dispersive X-ray detection (EDX), and X-ray diffractometry (XRD) to study the erosion of different kind of refractory materials by alkali metal carbonates [21,22,23,24,25,26,27,28]. The corrosion mechanism of refractory materials by sodium salts is studied from the viewpoint of slag and sedimentt; the anti-sodium corrosion performance of different types of refractory materials is compared, and the best material is selected. It was found that MgO-based spinel and Al_2_O_3_-based spinel refractories could be more suitable for alkali furnaces than the low-chromium Al_2_O_3_-Cr_2_O_3_ refractory [29]. Prigent et al. [30] investigated the erosion of siliceous refractories by a variety of corrosive materials, including CaSO_4_, K_2_SO_4_, Na_2_SO_4_, KCl, and NaCl, and found that the corrosion products contained albite.

In summary, many researchers have studied the corrosion behaviour and the degree of different refractories by sodium salts. However, alkali-containing organic waste liquids seriously corrode refractories, which causes the refractory material of incinerators to flake off. These problems prompted us to investigate the corrosion mechanism, especially from a thermodynamics perspective, to study the corrosion of refractories by sodium salts.

In this study, thermodynamic equilibrium calculations were performed using the integrated thermodynamic database FactSage to predict the Gibbs free energy of each reaction under different conditions and the possibility and priority of reactions between refractory components and sodium carbonate. A laboratory corrosion test, representative of corrosion in industrial operating conditions, was carried out to assess the characteristics of the different refractory materials that were subjected to corrosion by sodium carbonate. A comparison between the experimental and thermochemical calculation results provided insight into the direction and limit of the reactions in the multi-reaction system and on the influence of the reaction conditions on the corrosion process. Our objectives were to describe the thermodynamic mechanism by which sodium carbonate reacts with the mineralogical phases of the refractory minerals. The acquired knowledge can provide theoretical guidance to predictably control the corrosion of refractory materials.

## 2. Materials and Methods

### 2.1. Refractory Materials Preparation and Characterization

**Figure 2:**
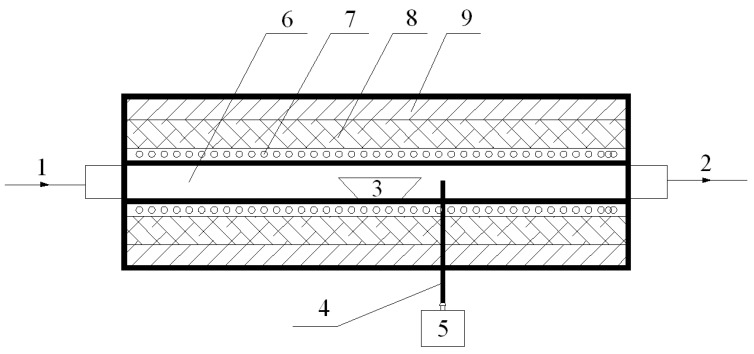
Schematic diagram of the high-temperature tube furnace. 1. air inlet; 2. air outlet; 3. sample holder (crucible); 4. thermocouple; 5. temperature control system; 6. tube reactor; 7. heating element; 8. refractory; 9. insulation.

In the investigations, six commercially available oxides and spinel refractory materials (numbered 1–6) were tested to study their corrosion by Na_2_CO_3_. The three oxide refractory samples are (1) Al_2_O_3_, (2) Fe_2_O_3_, and (3) Cr_2_O_3_, and the other three spinel refractory samples are (4) magnesium chromium (MgO·Cr_2_O_3_), (5) magnesioferrite (MgO·Fe_2_O_3_), and (6) magnesium aluminium (MgO·Al_2_O_3_). The three spinel refractories that were selected in this paper are commercially and commonly used refractories, which were synthesized in the laboratory. MgO (analytical purity) and Cr_2_O_3_ (analytical purity) were used as raw materials to synthesize sample 4. The MgO·Cr_2_O_3_ spinel was obtained by mixing Cr_2_O_3_ and MgO at a molar ratio of 1 and placing the mixture in a high-temperature tube furnace at 1500 °C for 4 h (Figure 2, Section 2.2 provides a detailed description). Sample 5 and sample 6 were prepared in the same manner. The XRD analysis and calculated RIR values reveal that the main mineral phase of samples 4–6 contained 90%, 83%, and 85% of MgO·Cr_2_O_3_, MgO·Fe_2_O_3_, and MgO·Al_2_O_3_, respectively (see composition in Table 1).

### 2.2. Thermochemical Experimental Apparatus and Procedure

The high-temperature corrosion tests were performed in a high-temperature tube furnace, as shown schematically in Figure 2, which consists of a quartz tube reactor and a temperature control section. The temperature of the tube furnace is automatically controlled by the temperature controller, which allows the temperature to vary between room temperature and 1700 °C.

**Figure 3:**
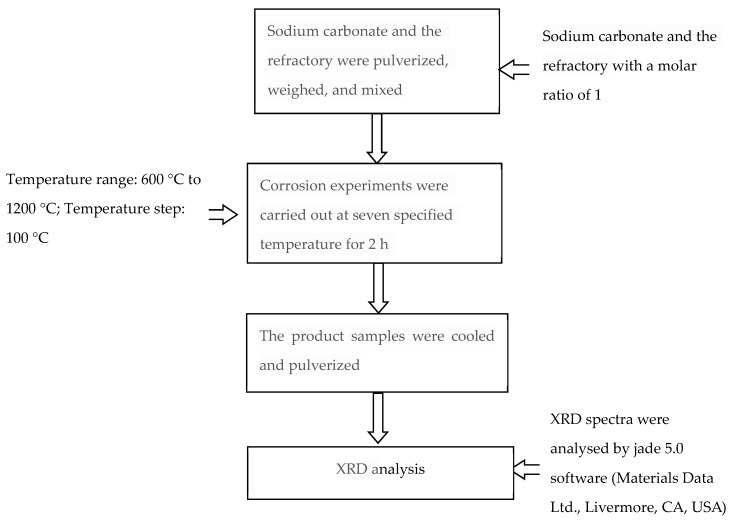
Flow chart of the thermodynamic experiment.

The refractory materials and Na_2_CO_3_ (analytical purity) samples (molar ratio of 1), with a diameter less than 200 mesh powders, were screened-dried to avoid hydration and placed in the crucibles. The crucibles were simultaneously heated in the high-temperature tube furnace at pre-determined temperatures (600 °C, 700 °C, 800 °C, 900 °C, 1000 °C, 1100 °C, and 1200 °C) under atmospheric pressure. Corrosion experiments were maintained for 2 h at the seven specified temperatures. When the experiment was completed, the tube furnace was cooled to room temperature, and the reaction product in the crucible was removed. After the high-temperature corrosion, the experiment reaction products were ground below 10 μm for a further analysis by XRD. The test flow chart is shown in Figure 3.

### 2.3. Analytical Determinations

The phase composition of the initial and reacted samples was determined by X-ray diffraction (XRD). The XRD patterns were recorded on an automated Shimadzu 7000S diffractometer (Shimadzu, Kyoto, Japan) using Cu Kα radiation and a secondary curved graphite monochromator. The anode voltage and current were 40 kV and 50 mA, respectively. The interval 10–70° (2θ) was scanned with a scan step of 5°/min.

### 2.4. Thermochemical Equilibrium Calculations

The thermochemical equilibrium calculations were performed with the FactSage software package (version 6.4, GTT Technologies, Aachen, Germany and Thermfact/CRCT, Montreal, QC, Canada). The FToxid, FTmisc, FTsalt, and FactPS thermodynamic databases were obtained by the evaluation and optimization of hundreds of molten metals, oxide liquid and solid phases, matte smelting systems, molten salt solutions, and aqueous solutions. The corrosion of refractory materials by Na_2_CO_3_ was considered to occur under isothermal and isobaric conditions in the thermodynamic analysis. The Gibbs free energy of reaction is often adopted to determine the direction and extent of a reaction at constant temperature and constant pressure. FactSage was used to calculate the Gibbs free energy and the phase equilibrium reaction thermodynamics between the refractory materials and Na_2_CO_3_ between 600 and 1200 °C.

## 3. Results and Discussion

### 3.1. FactSage Thermodynamic Calculations

#### 3.1.1. Decomposition and Synthesis Reactions of the Spinels

**Figure 4:**
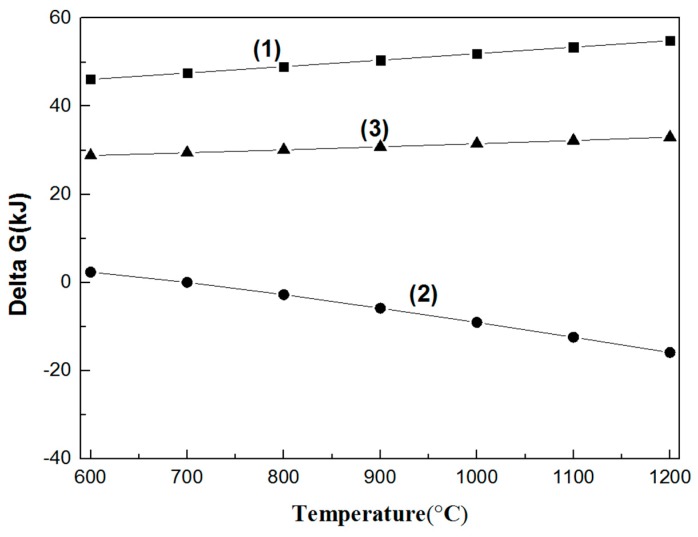
Gibbs free energy of the decomposition reaction of the three spinels in the 600–1200 °C temperature range. (1) MgO·Cr_2_O_3_ = MgO + Cr_2_O_3_; (2) MgO·Fe_2_O_3_ = MgO + Fe_2_O_3_; (3) MgO·Al_2_O_3_ = MgO + Al_2_O_3_.

**Figure 5:**
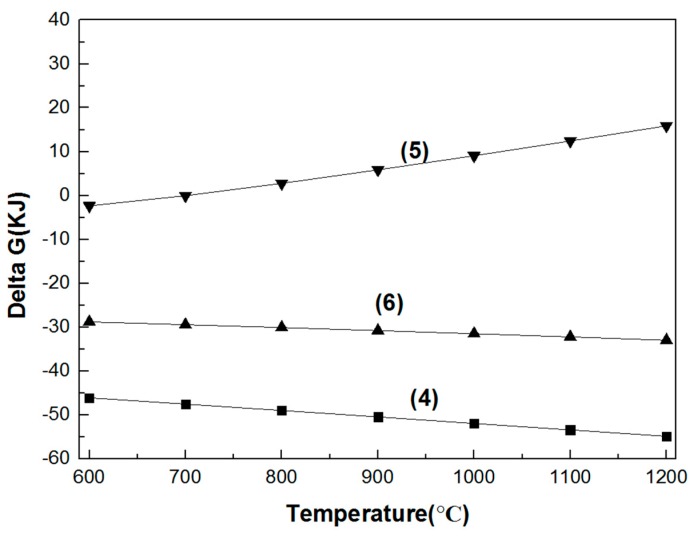
Gibbs free energy of the synthesis reaction of the three spinels in the 600–1200 °C temperature range. (4) MgO + Cr_2_O_3_ = MgO·Cr_2_O_3_; (5) MgO + Fe_2_O_3_ = MgO·Fe_2_O_3_; (6) MgO + Al_2_O_3_ = MgO·Al_2_O_3_.

The Gibbs free energy of the decomposition and synthesis reactions of the three spinels at temperatures from 600 to 1200 °C are shown in Figure 4 and Figure 5. The Gibbs free energies of the decomposition of MgO·Cr_2_O_3_ and MgO·Al_2_O_3_ are positive, while the Gibbs free energy of the corresponding synthesis reactions at high temperature is lower than that of MgO·Fe_2_O_3_. Thus, it is hard to decompose and easy to synthetize MgO·Cr_2_O_3_ and MgO·Al_2_O_3_ at high temperatures. The Gibbs free energy of the decomposition of MgO·Fe_2_O_3_ becomes negative with increasing temperature, illustrating the decomposition at high temperature.

#### 3.1.2. Reaction between the Six Refractory Materials and Na_2_CO_3_

**Figure 6:**
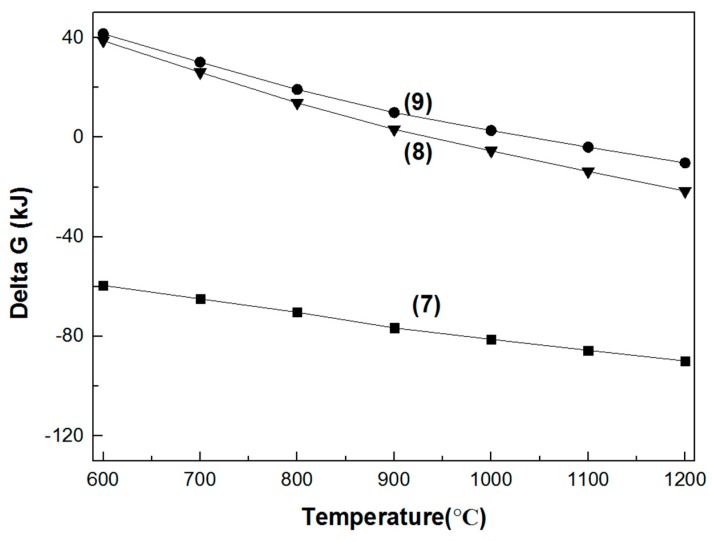
Gibbs free energy of the reaction between Na_2_CO_3_ and the three spinels in the 600–1200 °C temperature range. (7) Na_2_CO_3_ + 1/2MgO·Cr_2_O_3_ + 3/4O_2_ = 1/2MgO + Na_2_CrO_4_ + CO_2_; (8) Na_2_CO_3_ + MgO·Fe_2_O_3_ = Na_2_O·Fe_2_O_3_ + CO_2_ + MgO; (9) Na_2_CO_3_ + MgO·Al_2_O_3_ = MgO + Na_2_O·Al_2_O_3_ + CO_2_.

**Figure 7:**
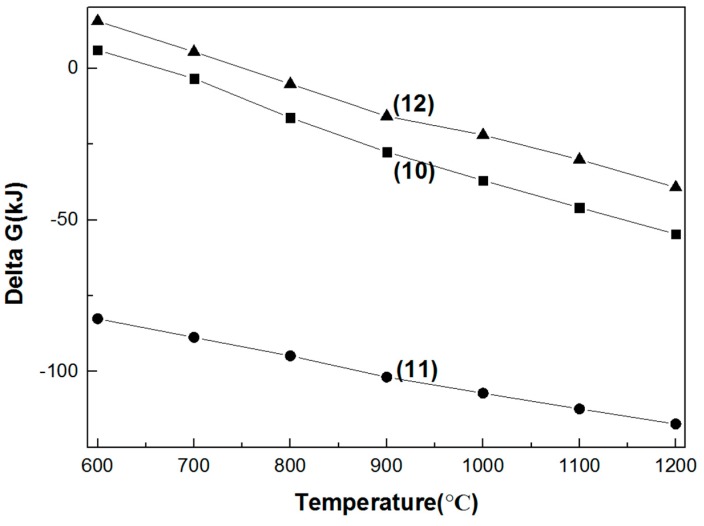
Gibbs free energy of the reaction between Na_2_CO_3_ and the three metal oxides in the 600–1200 °C temperature range. (10) Na_2_CO_3_ + Al_2_O_3_ = Na_2_O·Al_2_O_3_ + CO_2_; (11) Na_2_CO_3_ + l/2Cr_2_O_3_ + 3/4O_2_ = Na_2_CrO_4_ + CO_2_; (12) Na_2_CO_3_ + Fe_2_O_3_ = Na_2_O·Fe_2_O_3_ + CO_2_.

As seen in Figure 6 and Figure 7, with the increase in temperature, the Gibbs free energy of the reaction between the six refractory materials and sodium carbonate decreases. The Gibbs free energy of the reaction between sodium carbonate and MgO·Cr_2_O_3_ is negative at 600 °C; thus, we can conclude that the chromium-containing spinel reacts with sodium carbonate at low temperature. With the increase in temperature, the Gibbs free energy of the reaction between sodium carbonate and MgO·Fe_2_O_3_ and MgO·Al_2_O_3_ decreases. When the temperature is higher than 1000 °C, the Gibbs free energy is negative, indicating that these two spinels can react with sodium carbonate at high temperature. Figure 7 shows that the Gibbs free energy of the reaction between chromium oxide and sodium carbonate is very low, which indicates that the reaction proceeds at low temperature.

#### 3.1.3. Reaction of Intermediates

**Figure 8:**
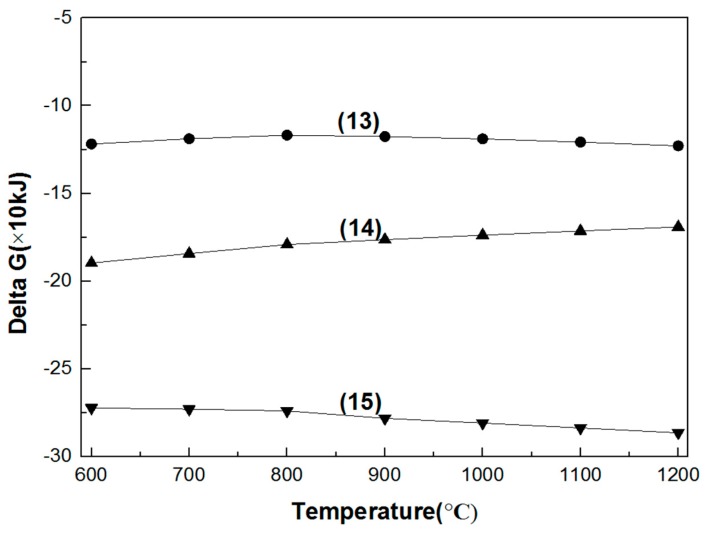
Gibbs free energy of the reactions between Na_2_CO_3_ and intermediate products in the 600–1200 °C temperature range. (13) Na_2_O·Fe_2_O_3_ + 1/2MgO·Cr_2_O_3_ + 3/4O_2_ = 1/2MgO + Na_2_CrO_4_ + Fe_2_O_3_; (14) Na_2_O·Cr_2_O_3_ + 3/4O_2_ = Na_2_CrO_4_ + 1/2Cr_2_O_3_; (15) Na_2_CO_3_ + Na_2_O·Cr_2_O_3_ + 3/2O_2_ = 2Na_2_CrO_4_ + CO_2_.

As seen Figure 8, in the temperature range of 600–1200 °C, the trivalent chromium-containing binary sodium salt is easily oxidized into the hexavalent chromium sodium salt in an oxidizing atmosphere. Moreover, sodium chromite is not stable, and it is converted to sodium chromate, regardless of the presence or absence of sodium carbonate. The Gibbs free energy is more negative in the presence of sodium carbonate, indicating that the presence of sodium carbonate is more conducive to the formation of a hexavalent chromium sodium salt.

### 3.2. Thermodynamics Experiments

#### 3.2.1. Effect of Temperature on the Reaction of Metallic Oxides with Sodium Carbonate

**Figure 9:**
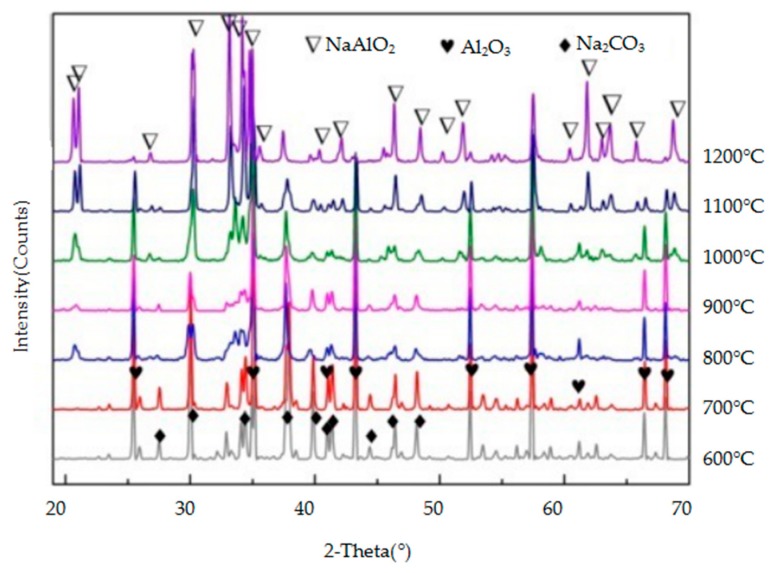
X-ray diffraction (XRD) patterns of the products of the reaction between Al_2_O_3_ and Na_2_CO_3_.

**Figure 10:**
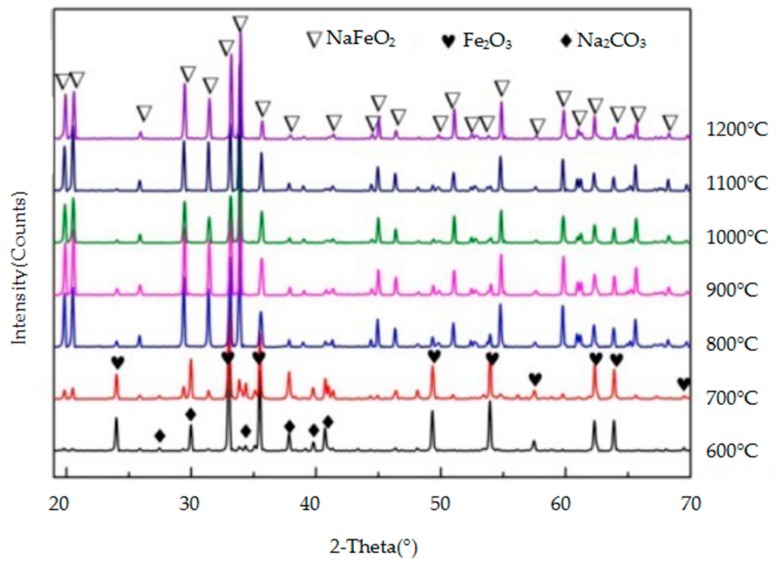
XRD patterns of the products of the reaction between Fe_2_O_3_ and Na_2_CO_3_.

**Figure 11:**
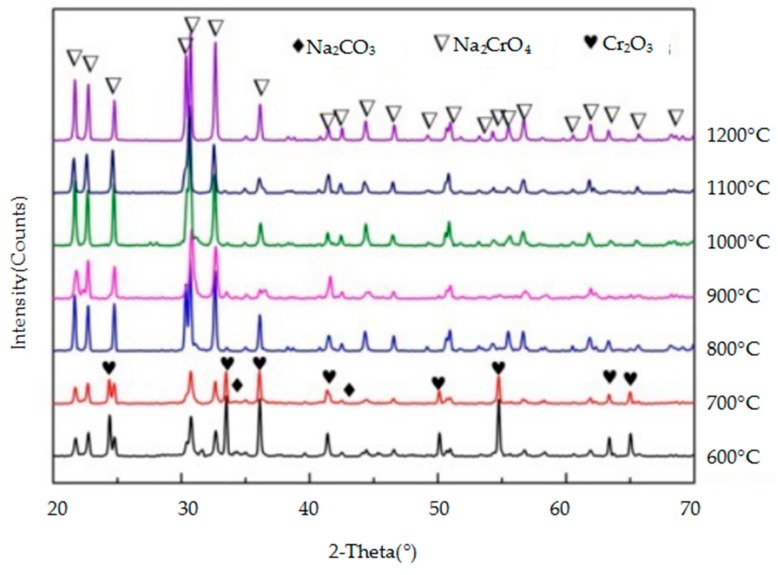
XRD patterns of the products of the reaction between Cr_2_ O_3_ and Na_2_CO_3_.

The crystalline mineral species in the reaction products of the three metallic oxides (Al_2_O_3_, Cr_2_O_3_, and Fe_2_O_3_) with sodium carbonate were identified by XRD and they are shown in Figure 9, Figure 10 and Figure 11, respectively. As the temperature increases, so does, the peak intensity of the products and the reaction between the metallic oxide and sodium carbonate is more favourable. When the temperature is lower (600–700 °C), the reaction between Al_2_O_3_ and Fe_2_O_3_ and Na_2_CO_3_ is not obvious, and the peak intensity of the products is lower. However, when the temperature reaches 800 °C, the progress of the two reactions is obvious, and the intensity of the NaAlO_2_ and NaFeO_2_ peaks is higher. Cr_2_O_3_ reacts with Na_2_CO_3_ at a lower temperature (600 °C) to produce Na_2_CrO_4_. From Figure 9, Figure 10 and Figure 11, it can be concluded that Cr_2_O_3_ reacts most easily with Na_2_CO_3_, followed by Al_2_O_3_ and Fe_2_O_3_.

#### 3.2.2. Effect of Temperature on the Reaction of the Three Spinels with Sodium Carbonate

**Figure 12:**
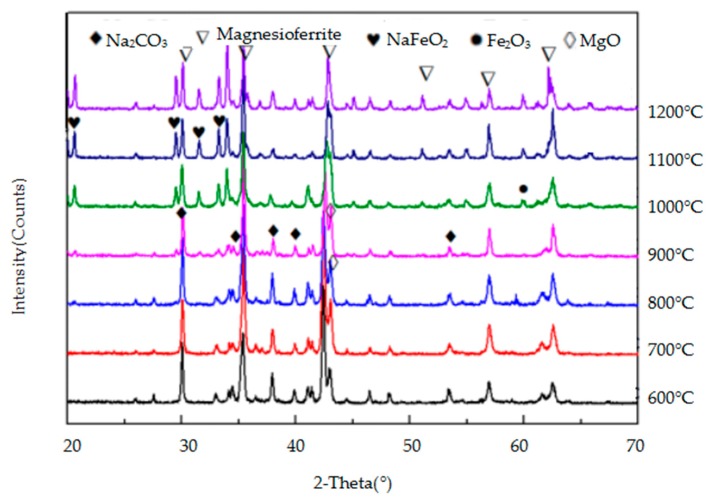
XRD patterns of the products of the reaction between MgO·Fe_2_O_3_ and Na_2_CO_3_.

**Figure 13:**
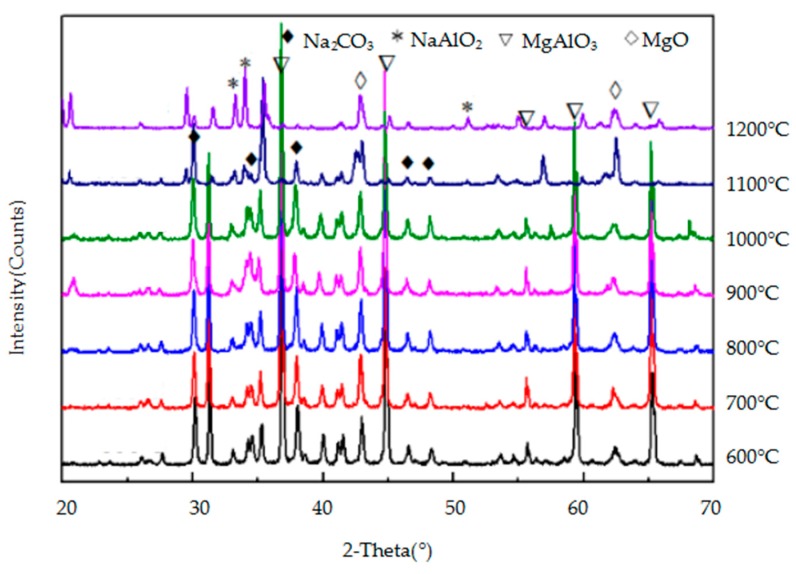
XRD patterns of the products of the reaction between MgO·Al_2_O_3_ and Na_2_CO_3_.

**Figure 14:**
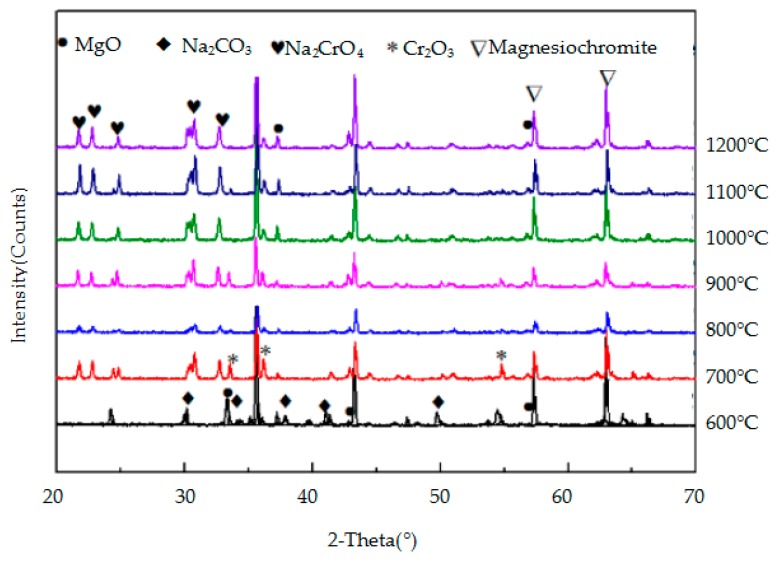
XRD patterns of the products of the reaction between MgO·Cr_2_O_3_ and Na_2_CO_3_.

The crystalline mineral species in the reaction products of MgO·Fe_2_O_3_, MgO·Al_2_O_3_, and MgO·Cr_2_O_3_ with Na_2_CO_3_ were identified by XRD and they are shown in Figure 12, Figure 13 and Figure 14, respectively. Figure 12 and Figure 13 show that when the temperature is lower than 1000 °C, MgO·Fe_2_O_3_ and MgO·Al_2_O_3_ barely react, and when the temperature reaches 1000 °C, a weak product peak appears. Moreover, the peak intensity of these two spinels remains high, indicating that MgO·Fe_2_O_3_ and MgO·Al_2_O_3_ do not easily react with sodium carbonate and are therefore highly corrosive to sodium carbonate. As seen in Figure 14, at 700 °C, the peak intensity of the reaction sample is very weak, and the peak intensity of the products is strong. As the temperature increases, the peak intensity of the reaction products further increases, indicating that MgO·Cr_2_O_3_ reacts very easily with Na_2_CO_3_ and it is therefore corroded. Therefore, MgO·Fe_2_O_3_ and MgO·Al_2_O_3_ have the stronger resistance to Na_2_CO_3_ corrosion, while MgO·Cr_2_O_3_ is the most easily eroded by Na_2_CO_3_. These findings on the reaction of the three spinels and sodium carbonate were also observed in the FactSage thermodynamic calculations discussed in Section 3.1.2 of this paper.

## 4. Conclusions

A combination of theory and experiment was used to study the corrosion of various components of refractories by sodium carbonate. The Gibbs free energy of reaction was calculated using the FactSage software to determine the ease of reaction, and the experimental products were analysed by XRD. The thermodynamic properties of the refractory materials and sodium carbonate were discussed.

(1) Spinel compounds in refractory materials are generally not susceptible to thermal decomposition reactions. The Mg-Cr and Mg-Al spinels are more difficultly pyrolysed and they have good thermal stability.
(2) The chromium-containing spinel reacts easily with Na_2_CO_3_ to form Na_2_CrO_4_. However, Na_2_Cr_2_O_4_ is not stable and can be converted to Na_2_CrO_4_ and Cr_2_O_3_. In the presence of Na_2_CO_3_, the dibasic sodium salt is more easily converted to Na_2_CrO_4_, indicating that the presence of sodium salts causes chromium-containing refractories to be more susceptible to corrosion, which shortens their service life.
(3) The difficulty of the reaction between the spinel and sodium carbonate is as follows: MgO·Al_2_O_3_, MgO·Fe_2_O_3_, and MgO·Cr_2_O_3_.
(4) Chromium oxide can react with sodium carbonate at a lower temperature of 600 °C and it has the worst corrosion resistance.

These results reveal the thermodynamic mechanism by which sodium carbonate reacts with the mineralogical phases of the refractory minerals. The results of the present work contribute to the use of refractory material in industrial waste liquid boilers. However, it should be noted that the corrosion mechanism of refractory materials by other alkali metals need to be further studied because of the complex composition of waste liquids.

## Figures and Tables

**Table 1:** Composition and mineral phase of the refractory samples determined by XRD analysis.

Sample	Mineral Phase	Content of the Main Mineral Phase	Manufacturer
1	Al_2_O_3_	analytical purity	Shanghai Macklin Biochemical Co., Ltd., Shanghai, China
2	Fe_2_O_3_	analytical purity, 99.0%	Shanghai Macklin Biochemical Co., Ltd., Shanghai, China
3	Cr_2_O_3_	analytical purity, 99.0%	Shanghai Macklin Biochemical Co., Ltd., Shanghai, China
4	Spinel (MgO·Cr_2_O_3_)	90 wt.% MgO·Cr_2_O_3_	self-made
5	Spinel (MgO·Fe_2_O_3_)	83 wt.% MgO·Fe_2_O_3_	self-made
6	Spinel (MgO·Al_2_O_3_)	85 wt.% MgO·Al_2_O_3_	self-made
